# The pharmacokinetics of epinephrine/adrenaline autoinjectors

**DOI:** 10.1186/s13223-021-00511-y

**Published:** 2021-03-08

**Authors:** Sten Dreborg, Harold Kim

**Affiliations:** 1 Department of Child and Adolescent Allergology, Women’s and Children’s Health, University of Uppsala, 751 85 Uppsala, Sweden; 2 Department of Medicine, Western University, London, Canada; 3 Department of Medicine, McMaster University, Hamilton, Canada

**Keywords:** Epinephrine, Autoinjector, Pre-filled syringe, Pharmacokinetics, T_max_, C_max_, Intramuscular, Subcutaneous, Area under the curve (AUC)

## Abstract

**Background:**

For a century, epinephrine has been the drug of choice for acute treatment of systemic allergic reactions/anaphylaxis. For 40 years, autoinjectors have been used for the treatment of anaphylaxis. Over the last 20 years, intramuscular epinephrine injected into the thigh has been recommended for optimal effect.

**Objective:**

To review the literature on pharmacokinetics of epinephrine autoinjectors.

**Results:**

Six studies assessing epinephrine autoinjector pharmacokinetics were identified. The studies, all on healthy volunteers, were completed by Simons, Edwards, Duvauchelle, Worm and Turner over the span of 2 decades. Simons et al. published two small studies that suggested that intramuscular injection was superior to subcutaneous injection. These findings were partially supported by Duvauchelle. Duvauchelle showed a proportional increase in C_max_ and AUC_0-20_ when increasing the dose from 0.3 to 0.5 mg epinephrine intramuscularly. Turner confirmed these findings. Simons, Edwards and Duvauchelle documented the impact of epinephrine on heart rate and blood pressure. Turner confirmed a dose-dependent increase in heart rate, cardiac output and stroke volume. Based on limited data, confirmed intramuscular injections appeared to lead to faster C_max_. Two discernable C_max’s_ were identified in most of the studies. We identified similarities and discrepancies in a number of variables in the aforementioned studies.

**Conclusions:**

Intramuscular injection with higher doses of epinephrine appears to lead to a higher C_max_. There is a dose dependent increase in plasma concentration and AUC_0-20_. Most investigators found two C_max’s_ with T_max_ 5–10 min and 30–50 min, respectively. There is a need for conclusive trials to evaluate the differences between intramuscular and subcutaneous injections with the epinephrine delivery site confirmed with ultrasound.

## Introduction

Epinephrine/adrenaline is the drug of choice for treatment of systemic allergic reactions/anaphylaxis [1]. Epinephrine has been used for about a century for treatment of acute allergic reactions, and has been recommended by authorities as the first-line treatment of anaphylaxis for nearly six decades [2]. The Swedish Medical Product Agency was the first regulator to advocate the use of epinephrine in 1964. It has been recommended as first choice for treatment of anaphylaxis by academic allergy societies [3, 4]. Epinephrine has been used for the treatment of anaphylactic events in the community using prefilled syringes for six decades [5] and as epinephrine autoinjectors (EAIs) for more than four decades [6]. For two decades, epinephrine injections have been recommended to be delivered intramuscularly (IM) into the mid antero-ventral thigh [7]. The evidence for IM injection in the mid antero-ventral thigh being superior to subcutaneous injection originates from the studies by Simons et al. [8, 9]. International recommendations have been to deliver EAIs into the mid antero-ventral thigh on the preferred side for the patient [1].

The aim of this paper is to evaluate the distribution of epinephrine after injection with syringe and needle and with EAIs as the importance of intramuscular injection has been questioned recently. Also, our findings may influence the design of future autoinjectors. Data presented in five studies on epinephrine pharmacokinetics identified [8–13] are summarized in Tables 1, 2. We will review the key aspects and variables from these studies.

**Table 1:** Pharmacokinetic studies of epinephrine, 0.3 or 0.5 mg, and syringe and needle. Comparison of injection in the middle part of the antero-lateral (m a-l) area of the thigh

Subject\Study	Simons et al.		Duvauchelle et al. [11]		Worm et al. [12]		
	1998 [8]	2001 [9]			Low STMD < 15 mm	Middle STMD 15–20 mm	High STMD > 20 mm
Number of pat.	9	13	18	18	12	12	11
Needle length	?	?	25.4 mm	25.4 mm	19.4 (12–30)	27.9 (2–40)	39.1 (30–40)
Gender	Children	Male, adults	Male, adults	Male, adults	M = 6; F = 6	M = 6; F = 6	M = 5; F = 6
Weight kg, Mean ± S.D./range	32 ± 3; 19.1–34.5	85 ± 5; 62–114	75 ± 10.5	75 ± 10.5	M/F 103.5/63.6	M/F 107.7/83.3	M/F 122.4/85.0
BMI kg/m^2^	–	36.6 ± 4.6; 20–64	23.3 ± 1.9; 18–26	23.3 ± 1.9; 18–26	M/F 32,4/21.3	M/F 32.2/28.1	M/F 36./31.4
Age years	8 ± 1; 4–12	18–35	31.5 ± 9.2	31.5 ± 9.2	M/F 36.2/46.0	M/F 34.5/38.8	M/F32.8/39.0
Site	?	Thigh, m a-l	Thigh, m a-l	Thigh, m a-l	Thigh, m a-l	Thigh, m a-l	Thigh, m a-l
Route	s.c.^a^	i.m.^a^	i.m.^a^	i.m.^a^	i.m.	i.m.	i.m.
Dose. mg	0.27 ± 0.04^b^	0.3	0.3	0.5	0.3	0.3	0.3
Plasma level analyzes hours	3 h	3 h	6 h	6 h	6 h	6 h	6 h
T_max_ 1, min.	34 ± 14 (5^c^–120)	10	11 ± 9; 5^d^	10 ± 8; 5^d^	40 (25–60)	45 (3–60)	50 (6–60)
T_max_ 2, min.	≈ 40^e^	?^f^	≈ 50^d^	≈ 50^d^	–	–	–
C_max_ 1 pg/ml	1802 ± 214	9722 ± 4.801	222.6 ± 185	401.2 ± 267	M/F 250/360	M/F 310/320	M/F 270/510
C_max_ 2 pg/ml	≈ 800^e^	≈ 1100^f^	402.2 ± 208	578 ± 251	–	–	–
No of C_max_	2?	1?	2	2	2	2	2
AUC_0-20_ h*pg/ml		–	38.5 ± 33.7	75.9 ± 52.6	*	*	*
AUC_inf_ h*pg/ml	610^3^ ± 13*10^3^		503.3 ± 201	777.2 ± 313	M/F 201/288	M/F 257/281	M/F 226/306

^a^Intended localization of the bolus; ^b^0.01 mg/kg, max 0.3 mg. 6/9 children received 0.3 mg, 1/9 children received 0.2, 1/9 0.23 and 1/9 0.24 mg ephedrine; ^c^In the s.c. group, 2/9 children and in the i.m. group, 6/8 children showed a T_max_ at 5 min. [8]; ^d^ Estimated from Figure 3 in [11]; ^e^ Estimated from Figure 1 in [8]. ^f^ According to Figure 1 in [9]

*AUC 6 min and AUC 15 min was measured but data not given

**Table 2:** Pharmacokinetic studies of epinephrine autoinjectors. Comparison of injection in the middle part of the antero-lateral (m a-l) or distal-ventral (d-v) area of the thigh

Subject\Study	Simons et al.		Edwards et al. 2013 [10]		Duvauchelle et al. 2018 [11]			Worms et al. [12]			Turner et al. [13]	
	1998 [8]	2001 [9]						Low STMD	Middle STMD	High STMD		
Brand	Epipen^®^	Epipen^®^	Epipen^®^	Auvi-Q^®^	Anapen^®^	Anapen^®^	Anapen^®^	Epipen^®^	Epipen^®^	Epipen^®^	Emerade^®^	Emerade^®^
Needle length, mm^j^	10.5	15	15	15	10.5	10.5	10.5	13–18 (20)	13–18 (20)	13–18 (20)	23	23
Number of pat.	8	13	135	67	18	12	18	12	12	11	12	12
Gender	Children	Male, adults	Male, adults	Male, adults	Male, adults	Female, adults	Male, adults	M = 6; F = 6	M = 6; F = 6	M = 5; F = 6	Adolescent M = 7; F = 5	Adolescent M = 7; F = 5
Weight kg	? 15–40	85 ± 5; 62–114	77.0 ± 11.5	77.0 ± 11.5	75 ± 10.5	78 ± 7.9	75 ± 10.5	M/F 103.5/63.6	M/F 107.7/83.3	M/F 122.4/85.0	61 41.8–76.4	61 41.8-76.4
BMI kg/m^2^	–	36.6 ± 4.6; 29–64	25.4 ± 2.7	25.4 ± 2.7	23.3 ± 1.9; 18–26	29.7 ± 1.9; 26–34	23.3 ± 1.9; 18–26	M/F 32.4/21.3	M/F 32.2/28.1	M/F 36./31.4	–	–
Age years	4–12	18–35	33.2 ± 6.3	33.2 ± 6.3	31.5 ± 9.2	33.3 ± 9.1	31.5 ± 9.2	M/F 36.2/46.0	M/F 34.5/38.8	M/F 32.8/39.0	15.4	15.4
Site	?	Thigh, m a-l	Thigh, m a-l	Thigh, m a-l	Thigh, m a-l	Thigh, d-v	Thigh, m a-l	Thigh, m a-l	Thigh, m a-l	Thigh, m a-l	Thigh m a-l	Thigh m a-l
Rote	i.m.^a^	i.m.^a^	i.m.^a^	i.m.^a^	i.m.	i.m.^a i^	i.m.	i.m.^a^	i.m.^a^	i.m.^a^	i.m.	i.m.
Dose mg	0.3	0.3	0.3	0.3	0.3	0.3	0.3	0.3	0.3	0.3	0.3	0.5
Plasma level analyzes hours	3	3	6	6	6	6	6	6	6	6	3	3
T_max_ 1, minutes	8 ± 2^c^ 5	10^f^	5–10^g^; 5^i^	5–10^g^; 5^i^	12 ± 7; 5^d^	14 ± 6; 15^d^	13 ± 7; 5^d^	9 (3–60)	10,5 (2–39)	30 (9–120)	–	–
T_max_ 2, minutes	≈ 20^e^	≈ 40^f^	25–30^8)^	25–30^h^	≈ 50^d^	≈ 30^d^	≈ 50^d^	–	–	–	–	–
C_max_1 pg/ml	2136 ± 351	12,222 ± 3829	520	486	377. ± 298 275^d^	440 ± 416 ≈ 250^d^	353.9 ± 283 ≈260^d^	M/F 400/640	M/F 480/520	M/F 420/630	–	–
C_max_2 pg/ml	1000^f^	≈ 6000^f^	≈ 300^h^	≈ 330^h^	≈ 250^d^	≈ 275^d^	≈290^d^	–	–	–	–	–
No of C_max_	1?	2	2	2	2	2?	2	2	2	2	2	2
AUC_0-20_ h*pg/m^k^	–	–	–	–	69.3 ± 54	55.9 ± 45	68.4 ± 50				–	–
AUC_0-t_ h*pg/ml	–	–	466	536	–	–	–	M/F 273/288	M/F 281/209	M/F 263/311	–	–
AUC_inf_ h*pg/ml	108,000 ± 18,000	–	583	724	459.2 ± 129	677.7 ± 161	473.1 ± 139	–	–	–	–	–

^a^Intended localization of the bolus; ^c^In the s.c. group, 2/9 children and in the i.m. group, 6/8 children showed a T_max_ at 5 min. [8]; ^d^ Estimated from Figure 3 in [11]; ^e^ Estimated from Figure 1 in [8]. ^f^ According to Figure 1 in [9]; ^g^ Approximately according to Table 3 in [10]; ^h^ According to Figure 2 in [10]; ^i^ s.c. in 10/12 women, i.m in 2/12 women as localized by ultrasound [10]. ^j^ The variation 5 mm for Epipen^®^, not known for Anapen^®^ [11, 27]; ^k^ Worm [12]: AUC registered for 6, 15 and 30 min, but data not reported

## Selection of studies

A systematic literature search was performed in May 2020, using PubMed and the keywords “anaphylaxis” and “pharmacokinetic” and “epinephrine” and “autoinjector”. Different combinations of these search terms were used as well.

Six publications were found addressing the pharmacokinetics of epinephrine using EAIs [8–12, 13]. All of the papers are presented in Tables 1, 2. Sclar [14] compared Auvi-Q^®^ and Epipen^®^, without publishing brand specific data. Simons et al. described the effect of injection of epinephrine by syringe and needle and by a high pressure EAI (HPEAI) Epipen^®^, in children in 1998 [8] and in adults in 2001 [9]. Since then, intramuscular injection in the mid antero-ventral thigh has been the general recommendation [1]. Song [15] speculated that epinephrine injection with an HPEAI into the subcutaneous tissue would propel the drug through the fascia into the muscle. This hypothesis has been supported by injection into ballistic gel. Diacono et al. [16] injected into the subcutaneous tissue of pigs. He confirmed that the fluid did not penetrate from the subcutaneous space through the fascia into the muscle, contradicting the hypothesis of Song. Duvauchelle et al. [11] published new data on the deposition of the epinephrine bolus. They visualized the location of the fluid bolus of epinephrine by ultrasound in humans. In ten of twelve obese and overweight women who received the injections with an HPEAI, Anapen^®^, the bolus was located in the subcutaneous tissue at the tip of the injection needle not penetrating the muscle. Recently, Worm et al. [12] reported on the pharmacokinetics and pharmacodynamics of Epipen^®^ and syringe and needle injections in groups of women and men with different degrees of skin to muscle distance. In addition, Turner et al. [13] reported on the pharmacokinetics of epinephrine autoinjector use in adolescents at the American Academy of Allergy Asthma and Immunology Annual Meeting in 2020.

## Overview of patients

The following is a summary of the patients included in the published studies.

Intramuscular injections with syringe and needle were performed in 93 patient divided into several groups: Nine children in one group [8], 66 adult men in six groups [9, 12], and 18 women divided into three groups.

In groups injected with EAIs (Table 2), there were 8 children in one group, 12 women in one group and 251 adult men were included in five groups. A total of 271 individuals with 8 to 135 individuals per group. Furthermore, three sex-mixed groups of adults with 18 females and 17 men and seven adolescent boys and five adolescent females, formed three and two groups, respectively [13].

The adult men in Simons et al. [9] study were on average obese with mean BMI 35 kg/m^2^. The men included by Edwards et al. [10] and Duvauchelle et al. [11] had normal weight and BMI. The women included in the trial by Duvauchelle et al. [11] were overweight or obese, BMI mean 29.7 kg/m^2^, range 26–34 kg/m^2^. Worm [12] included six men and six women with short skin to muscle distance (STMD), middle STMD and high STMD, respectively, with three different weight and BMI characteristics.

The patients included in the studies were volunteers. All of the subjects had normal heart rate, blood pressure and were not experiencing allergic reactions at the time of the studies. Importantly, in real world practice, autoinjectors are recommended for use in patients with severe systemic allergic reactions or anaphylaxis. These patients are suffering from acute allergic symptoms (shortness of breath, throat tightness) and signs (low blood pressure, wheezing, angioedema, urticaria). Epinephrine is the only readily available drug that may reverse all these changes.

In summary, the patients included in the studies performed to this time, consisted of small groups of subjects with fewer children and females. Some of the included men were obese, but most had “normal” BMI. Most female subjects were obese or overweight. All subjects were symptom-free during the studies (Tables 1, 2).

## Injection site and route

**Fig. 1:**
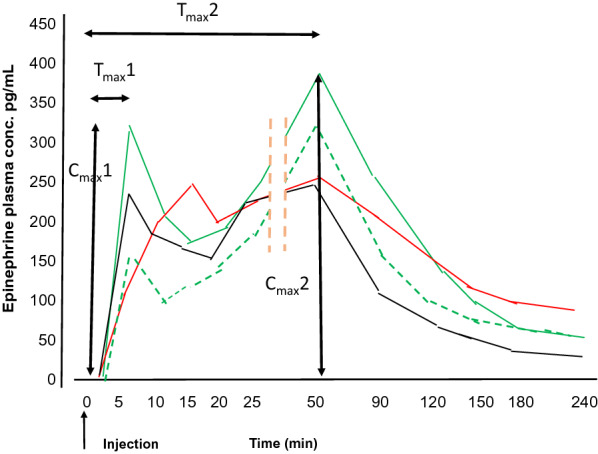
The group mean epinephrine plasma concentrations after injection of epinephrine: Injection with syringe and 1 inch needle in normal adult men, 0.3 mg green broken line, and 0.5 mg, green line, both with C_max_1 at T_max_1 5 min. Injection with high pressure epinephrine autoinjector, Anapen^®^, in normal adult men, 0.3 mg black line with C_max_1 at T_max_1 5 min and in obese adult women red line with C_max_1 at T_max_1 15 min. Ten of 12 women received subcutaneous injection Modified after Duvauchelle et al. [11]

Injections of epinephrine were made into the mid antero-ventral thigh, with two exceptions, Simons et al. [8], who injected children (n = 9) an individualized dose of 0.01 mg/kg body weight (mean 0.27 mg) of epinephrine with syringe and needle, or with Epipen^®^ 0.3 mg, into an unidentified anatomical location. Duvauchelle et al. [11] intended to inject the Anapen^®^ IM in the central inferior part of the femoralis muscle of 12 obese or overweight women. However, the men were injected in the mid antero-ventral thigh. Duvauchelle et al. [11] determined the depth of the injection bolus by ultrasonography, and found that 10/12 obese women received epinephrine subcutaneously, Fig. 1. Therefore, the red curve in Fig. 1 illustrating the plasma concentrations of epinephrine is a combination of the plasma concentrations after ten subcutaneous and two intramuscular injections, since all of the subjects did not receive subcutaneous injections. Turner et al. [13], Edwards et al. [10] and Worm et al. [12] used the mid antero-ventral thigh. Duvauchelle et al. [11] and Turner et al. [13] documented the presence of the epinephrine bolus in the intended site in the muscle by ultrasonography.

## Needle length

In recent years, we have published a number of papers, based on the original studies by Dr. Harold Kim’s group [17–19]. The aim has been to find out if present or future EAIs have the ability to deposit epinephrine in the intramuscular compartment of the vastus lateralis [20–24]. The outcome of these studies is that high pressure EAIs with shorter needles increase the risk of subcutaneous injection. Low pressure EAIs with longer needles are less likely to lead to subcutaneous injections due to longer needle length and are less likely to hit the bone due to less pressure required to trigger the device [23]. The length of EAI needles varies between brands and even within batches from the same manufacturer. Thick clothing increases the risk of subcutaneous injection and decreases the risk of intraosseous injection [22]. Obese individuals need longer needles (especially obese women) than the needles currently available in EAIs to reach the muscle [24]. Release of the spring of the autoinjector with low pressure on the EAI and minimal variation in approved needle length favors intramuscular injection. There are some studies supporting and others contradicting our findings. The different outcomes are likely due to minor differences in methodology.

In the Pink Book [25], the Centers for Disease Control and Prevention (CDCP) recommends a 1 to 1¼ inch (25.4–33.5 mm) needle for injection in the vastus lateralis in older children and adolescents. The recommendations of CDCP are given in Table 3. Recently, the Cochrane Database of Systematic Review recommended a 25 mm 23–25 G needle for intramuscular injection in children and adolescents [26].

**Table 3:** CDCP recommendations for for needle length for intramuscular injection

Gender				Needle length	
Male		Female		Inch	mm
lb	kg	lb	kg		
< 130	< ≈ 60	< 130	< ≈ 60	5/8–1	15.8
130–152	≈ 60–70	130–152	≈ 60–70	1	25.4
153–260	≈ 70–118	153–200	≈ 70– ≈ 90	1–1½	25.4–38.1
> 260	> ≈ 120	> 200	> ≈ 90	1½	38.1

In older children and adolescents, 1 to 1¼ inch (25.4–33.5 mm) needle for injection in the vastus lateralis

In their three groups, Worm et al. [12] used syringe with varying needle lengths depending on the STMD, from 12 to 40 mm, aiming at giving intramuscular injection. The other investigators probably used a 25 mm needle in their studies (Tables 1, 2).

The length of the needle of Anapen^®^ (Duvauchelle et al. [11]) has been stated to be 10.5 mm [11]. Epipen^®^ has an exposed needle length of 15.5 mm (5 mm variation, 13–18 mm [27]) used in the studies by Simons et al., Edwards et al. [10] and Worm et al. [12]. Emerade^®^ 0.3 mg and 0.5 mg have a 23 mm (1.5 mm variation) long needle (Turner et al. [13]).

## Pressure on the autoinjector

There are high pressure and low pressure autoinjectors. Recently, we showed that high pressure mainly compresses the muscle and not the subcutaneous tissues [28]. Therefore, low pressure on the EAI allows the use of longer needles that in turn favors intramuscular injection [23]. However, at this time there are no studies assessing serum epinephrine levels using the pressures on the probe/EAI specified by manufacturer for batch release and confirming the location of the injected bolus. In our studies, minimal pressure was applied to the ultrasound probe to mimic the pressure applied to a low pressure autoinjector, Emerade^®^, and about 8 lb or 35 N to the probe to mimic the pressure applied for release of the needle of high pressure EAIs (Epipen^®^, Jext^®^, Auvi-Q^®^, and Anapen^®^ etc.). The low pressure is now defined as 2–8 N and the high pressure to (EpiPen^®^) 8.5–35 N.

## Force induced by the autoinjector spring

Worm et al. [12] reported on an early C_max_, even though Epipen^®^, with a short needle, likely resulted in subcutaneous injections in one of their groups. They hypothesize that the strong spring of the Epipen^®^ “may enable the propulsion of epinephrine beyond the subcutaneous fat layer or promote greater contact between the injectate and the vascular bed, resulting in a greater dispersion and systemic uptake …”. The European Medicines Agency [29], EMA, hypothesize that there will be less uptake in the subcutaneous tissue due to that possible dispersion. Worm et al. [12] did not localize the injection bolus by ultrasound as did Duvauchelle et al. [11] and Turner et al. [13]. So in the Worm et al. study, subcutaneous delivery was not confirmed but assumed on pre-injection ultrasound measurements.

Currently there are no data on the true force induced by the spring of autoinjectors. Therefore, the hypothesis of Worm et al. [12] and the EMA [29] remain speculative.

## Localization of the injection bolus

The majority of data is not based on confirmed intramuscular or subcutaneous injections. Only Duvauchelle et al. [11] and Turner et al. [13] present data on the true localization of the injection bolus. And only Turner et al. [13] present group data based on only intramuscular injections. Simons injected obese men (BMI 29–64 kg/m^2^) with Epipen^®^ with a 15 mm (13–18 mm) exposed needle [21]. It is likely that some (or even most) of the injections were delivered subcutaneously, since their BMI was 36.6 kg/m^2^ range 29–64 kg/m^2^. However, the injections were considered to be delivered intramuscularly in the study. Duvauchelle et al. [11] defined injections as intramuscular or subcutaneous by localizing the injection bolus by ultrasound. One man and 10/12 women intended to be given intramuscular injections were shown to, in reality, have received subcutaneous injections. Turner et al. [13] included only confirmed IM injections in the analysis [13]. Duvauchelle et al. [11] combined the data from women who received intramuscular and subcutaneous injections in the same analysis and so the same plasma level curves. This may have led to lower epinephrine C_max_ levels than if all had received IM injections with similar T_max_1.

## Methods for determination of plasma levels of epinephrine

The methods for determination of plasma levels of epinephrine differed between investigators and laboratories. The variation was up to 60 times between studies. For unknown reasons, the highest plasma levels of all studies were found in a group of obese males [9]. These males had five times higher levels of plasma epinephrine than small children [8] after injection of the same dose of epinephrine at 0.3 mg. The groups of Duvauchelle et al. [11], Worm et al. [12] and Edwards et al. [10] reported similar plasma levels. However, these levels were much lower than those reported by Simons et al. [8, 9]. Methods for determination of concentrations of analytes using a known analyte as standard, should obtain similar results in similar subjects. The issue of methods for determination of epinephrine in plasma will not be discussed further in this review. However, data from Simons [8, 9] and the other investigators cannot be compared in the same figures due to the significant differences in reported serum levels.

We think, in order to allow comparison between studies, all data should be reported as pg/ml [8–12] or nM/ml [13].

## Plasma concentrations, C_max_1 and C_max_2 at T_max_1 and T_max_2

The plasma levels were measured at short intervals for 3 h by Simons et al. [8, 9] and Turner et al. [13] and for 6 h by Edwards et al. [30], Duvauchelle et al. [11], and Worm et al. [12].

The plasma concentration of epinephrine usually showed two peaks (C_max_) that are denoted as C_max_1 and C_max_2 at the times T_max_1 and T_max_2. Worm et al. [12] reported one C_max_. However, on reviewing the majority of the serum level curves, there are normally two peaks. The mean plasma epinephrine curves that included data from all individuals in a group who may have quite different absorption due to different injection sites (subcutaneous and IM) are flattened and C_max_ is even eradicated. This was seen in the group of adult obese men (BMI 29–65) injected by Simons et al. with 0.3 mg of epinephrine with syringe and needle [9]. Since the majority of groups had two group C_max_, we assume two C_max_ is the normal response to epinephrine injection in the subcutaneous and IM tissue. The reason for two C_max_ may be initial absorption, then vasoconstriction induced by epinephrine and then C_max_2 that occurs after increased absorption after vasodilatation. The C_max_1 was lower than the C_max_2 in most studies.

All data are presented as group mean data, with standard deviations and/or ranges. No data for individual subjects are presented, except for the number of children [8] injected subcutaneously and intramuscularly with 0.3 mg epinephrine.

T_max_1 at 5 min was obtained in 2/9 children who received subcutaneous injections and in 6/8 children who received an intramuscular injection. Those injected intramuscularly had a T_max_1 of subcutaneously had a mean T_max_1 of 34 min, range 5–120 min. To understand the kinetics, inspection of individual data and confirmation of the site of the injection bolus is needed.

All groups had a mean T_max_1 that varied between studies and groups. The constructed mean curves typically demonstrated a mean T_max_1 at 5 min. Only Edwards et al. [10] report on the individual variation in T_max_1 (Table 4). Many patients had a T_max_1 more than 30 min. This may have been due to subcutaneous injections. It has been shown that Epipen^®^ and Auvi-Q^®^ have a high risk of subcutaneous injection and the location of the epinephrine injections were not documented in this study. Furthermore, Worm et al. [12] injected individuals with a skin to muscle distance, STMD, more than 15.5 mm with Epipen^®^ with 15.5 mm needle. Therefore, it is possible that many injections were given SC.

There was a second T_max_, T_max_2, that occurred 20–50 min after injection. In most cases the C_max_2 peak was extracted from the figures of the papers (Fig. 1). In the study by Simons et al. in adult men [9], the T_max_2 was not present when epinephrine was injected by syringe and needle, but present after injection with Epipen^®^, possibly due to varying individual T_max_2 in each individual leading to a flattened curve without a clear T_max_2. The C_max_2 was not obvious in this same trial [9]. Worm et al. [12], report on C_max_. From their group curves, there is no clear C_max_1. This may be due to measurements not taken frequently enough to identify this early peak. We suggest that the frequency of epinephrine measurements be taken in a similar manner in the first 30–60 min as completed by Duvauchelle [11]. The T_max_ of their reported C_max_ was often 30 to even 60 min, indicating they have identified the C_max_2, Table 3. Worm et al. [12], presented their data in a method that makes it difficult to interpret. They showed the most variation in plasma epinephrine level occur within the first 5–15 min after injection compared to other studies. Duvauchelle et al. [11] used a more detailed presentation of the variation during the first hour that allows easier interpretation.

## Area under the curve

The area under the curve, AUC, was determined in three ways: AUC_0-20 min_ (AUC during the first 20 min), AUC_0-t_ (AUC from injection to last determination of the plasma level), and AUC_0-inf_ (AUC to infinity). AUC_0-inf_ was calculated to the theoretical end of epinephrine plasma concentration above the background levels.

The area under the curve was estimated by all authors. Simons et al. determined the AUC only in children [8] and only as AUC_inf_.

Edwards et al. [30] and Duvauchelle et al. [11] had similar AUC_inf_, 500 times lower than those of Simons et al. [8, 9], with a mean plasma level between 200 and 700 pg/ml, with pronounced inter-individual variation. Furthermore, Duvauchelle et al. [11] reported on AUC_0–20 min_ and Edwards et al. [30] on the AUC_0-t_. EAIs are indicated in subjects with the risk of immediate, severe, systemic allergic reactions. The most severe cases show life-threatening symptoms within minutes after exposure to the allergen(s). Therefore, we hypothesize, AUC_0–5 min_ (AUC from injection to 5 min), AUC_max5_ or AUC_0-10_ would be the most important indirect indicator of efficacy when assessing serum levels. Overall, the AUC_0–20 min_ is the best estimate of AUC that was presented in this group of studies. However, AUC_0–20 min_ was only about 10% of the AUC_inf_ for the studies where data was available. No authors measured the AUC to the C_max_1 or the AUC_0–5 min_.

## Group means

The publications by Simons et al. [8, 9], Duvauchelle et al. [11] and Edwards et al., [10] present figures with group data. When there was no patient level data on the group C_max_ in the text or tables in the studies, the data presented in this paper in Tables 1, 2, have been extracted from the figures of the original publications [8–11, 12, 13]. This is indicated with question marks in Tables 1, 2 to note some aspect of uncertainty.

Using group means as the only method of analysis may introduce major issues when analyzing the plasma levels. Individual data is preferred because variable results from individuals may eradicate or neutralize each other flattening or moving the C_max_1 and 2. Thus, an injection intended to be intramuscular that is given subcutaneously, or the opposite, may influence the serum levels flattening any peak that is expected with C_max_1 or 2. One possible example is the study by Simons et al. In the pediatric study, Simons et al. [8] found the T_max_1 after injection with syringe and needle to be 34 min. This relatively late T_max_1 may have occurred in this study due to some of the participants having a T_max_1 of up to 120 min (range 5–120 min). With a large range in T_max_1, the C_max_1 may be blunted. The T_max_ in this study is not comparable to the T_max_ noted in the other studies reviewed in this paper. It is possible that the T_max_ reported by Simons et al. may actually have been the T_max_2. We believe that only inspection of the original individual patient data, and or repetition of the experiments, can resolve this issue. Two of nine children who received subcutaneous injection had a T_max_1 of 5 min. That means that some children who were documented to be injected subcutaneously had a rapid absorption. The absorption was as rapid as in six children in the group who received injection with Epipen^®^ with a T_max_1 of 5 min. It is not clear if this rapid absorption was due to intramuscular injection or whether subcutaneous absorption occurred as rapidly as intramuscular.

In the study in adult men by Simons et al. [9] there was one T_max_ in the group injected with syringe and needle, but two T_max_ after five and forty minutes in those who had received injection with Epipen^®^. T_max_1 and 2 are both present in all the group-based curves presented by Duvauchelle et al. [11] and Edwards et al. [10]. The most probable reason for the lack of two T_max_ is that, in the study of Simons et al. [9] is that some injections with Epipen^®^ were not given intramuscularly but subcutaneously [21, 24]. This may have led to flatting of the plasma concentration curve (see above).

Today, we know that especially obese subjects have a high risk receiving subcutaneous injections when using Epipen^®^ [24]. In the group of adult men used by Simons et al. in 2001 [9], BMI was very high, maximum 65 kg/m^2^, and the weight was up to 114 kg (249 lb). In our previous paper on the influence of BMI on the risk for subcutaneous injection, we found that the majority of obese adults and many over-weight and normal-weight adults have the risk of subcutaneous injection using Epipen^®^ or Auvi-Q^®^ [24].

Adult men [9] weighing about three times more than the children showed six times higher plasma concentration than the children did [8, 9]. This is despite volumes of distribution that must be much larger in adult men. These higher plasma levels were unexpected findings when reviewing the results of the studies. Since the data by Simons et al. were different in the two trials, much higher than the plasma levels found by Edwards et al. [10], Duvauchelle et al. [11], and Worm et al. [12] and not presented in a way that made it possible to easily extract individual patient data, we refer the reader to the original articles [8, 9].

Duvauchelle et al. [11] performed ultrasound localization of the bolus after injection with Anapen^®^. They found that 10/12 women received a subcutaneous injection rather than the intended intramuscular injection.The EAI used, Anapen^®^, has a short needle length (mean needle length 10.5 mm). Using Anapen^®^ 0.3 mg, C_max_1 had a T_max_1 of 15 min, as opposed to the injections of the same amount of epinephrine with a syringe with 1-inch (25.4 mm) needle that had a T_max_1 after 5 min (Fig. 1). Immediate injection of epinephrine is important for improving the clinical outcomes in anaphylaxis. It is more likely that obese women similar to those in the study by Duvauchelle et al. [11], have a high risk of subcutaneous injection even with Epipen^®^ [24], The difference in T_max_1 may explain why obese women, with rapidly progressing symptoms, are more likely to die from anaphylaxis even when an EAI with shorter needle length and lower dose is given [31–35]. Actually, Pumphrey reported that [32] the autopsy of a young girl, who died from anaphylaxis, despite two Epipen^®^ injections showed that the puncture needle channel was 16 mm, the distance from skin to muscle 22 mm. Duvauchelle et al. [11] and Turner et al. [13] used ultrasound to confirm injection into the IM space.

Duvauchelle et al. [11] and Turner et al. [13] showed that increasing the dose of epinephrine from 0.3 mg to 0.5 mg increased the C_max_1 and C_max_2 (Fig. 1). Duvauchelle et al. [11] showed that even the AUC_0-20_ and the AUC_inf_ increased proportionally.

Edwards et al. [10] grouped Auvi-Q^®^ and Epipen^®^ data separately to compare the two brands. This study showed two T_max_ at approximately five and 30 min with C_max_1 and C_max_2 about 500 and 300 pg/ml, respectively. The AUC was similar for both brands and also similar to that of Duvauchelle et al. [11]. All but one experiment used the same amount of epinephrine, 0.3 mg, Auvi-Q^®^ and Epipen^®^ showed similar group mean curves and Auvi-Q^®^ and Epipen^®^ have the same needle length. Since Edwards et al. did not document the site of the epinephrine bolus our hypothesis is that the T_max_1 reported in Table 3 in some cases were due to subcutaneous injection. The mean curves by Edwards [10] appear smooth, but are probably blunted by some individuals with very late C_max_ (Tables 1, 2 and 4) due to the subjects in the study likely receiving subcutaneous injections.

In summary, group means when assessing epinephrine serum levels are somewhat misleading because there is large inter-individual variation in epinephrine absorption causing eradication or flattening of the group plasma concentration curves. This likely occurs in most published studies because the site of the epinephrine bolus is not documented to be intramuscular or subcutaneous. Only Duvauchelle et al. [11] and Turner et al. [13] present data from definite intramuscular or subcutaneous injections of epinephrine.

**Table 4:** The number of patients (%) with C_max_1 after different T_max_1

Group	Auvi-Q^®^ 1	Auvi-Q^®^ 2	Epipen^®^
Minutes	n 69 (%)	66 (%)	67 (%)
3–9	31 (45)	30 (43)	24 (36)
0–15	40 (58)	41 (61)	29 (43)
15–27	7 (10)	4 (6)	9 (13)
27–39	10 (14)	13 (20)	15 (22)
39–60	12 (17)	8 (12)	14 (21)

A majority of patients had a T_max_1 less than 15 min, i.e. epinephrine reached the circulation in time to counteract the effects of general allergic reactions/anaphylaxis. A majority of these patients reached a C_max_1 before 10 min post injection. The same patients were injected once with Epipen^®^ and twice with Auvi-Q^®^. Modified after Edwards [10] Table 3. Edwards gave the time in decimals of an hour; we modified to minutes and grouped data into fewer groups

## Summary of findings of the studies

Based on two landmark studies, Simons et al. [8, 9] proposed that intramuscular injection is superior to subcutaneous injection two decades ago. However, the data published by that group does not fully support this conclusion. Chawdbury and Meyer identified some of the potential flaws from these studies as we have identified above [36]. But they did not highlight the unexplained discrepancies in plasma epinephrine concentrations. The significant difference between the subcutaneous groups and the presumed intramuscular groups (Epipen^®^) may have occurred due to the variability in absorption of a few patients with very long T_max_1. Even the inter-individual variation in absorption and distribution may have influenced the lack of a C_max_2 after injection with syringe and needle. Furthermore, adult men weighing about three times more than the children showed six times higher plasma concentration than the children showed. For these reasons, in retrospect, the conclusions from Simons et al. [8, 9] studies are questionable.

Edwards et al. [10] compared two brands of EAIs with similar needle length and the same dose of epinephrine, showing similar group mean plasma levels T_max_ and C_max_. This study showed that the devices for these products lead to similar delivery of epinephrine. However, we cannot confirm that the epinephrine was delivered to the IM or subcutaneous tissues. Therefore, the data cannot be used to contribute to our understanding of the pharmacokinetics of autoinjector delivered epinephrine.

The hypothesis of Simons et al. [8, 9] that intramuscular injection causes a more rapid absorption than does subcutaneous injection was partially supported by Duvauchelle et al. [11]. They also showed that after injection with an autoinjector with an inadequately short needle length for IM delivery, the bolus remains at the tip of the injection needle in the subcutaneous tissue and does not propel through the fascia into the muscle. Furthermore, Duvauchelle et al. [11] showed that increasing the injected dose, from 0.3 to 0.5 mg, increased the C_max_1, the C_max_2 and the AUC_0-20_, proportionally.

Turner et al. [13] confirmed that increasing the dose from 0.3 to 0.5 mg of epinephrine and using an adequate needle length for IM delivery, increased the plasma levels of epinephrine and the AUC significantly. The confirmed increased physiologic changes with the higher dose of epinephrine may be clinically important in the treatment of anaphylaxis. However, in the data presented by Worm et al. [12], the epinephrine levels were measured less frequently than the Duvauchelle [11] paper in the first 10–15 min after the injection which makes it difficult to document as accurate C_max_1/T_max_1.

## Practical use of EAIs

The aim for the use of EAIs is to be injected as soon as early signs and symptoms of anaphylaxis appear. Therefore, any improvement of the function and pharmacokinetics of the EAI is of questionable value if it is not injected immediately when required. The patient can be alone or with their family, at school or with friends. Many of these people are not likely going to be familiar with the use of EAIs and will often be afraid of giving injections. The European Medicines Agency, EMA, prescribes that all companies should offer EAI training devices to improve adherence with EAI use. However, the training devices still do not reduce the fear of the real needle injection. In the USA, schools are obliged to keep an EAI available. If all adults and school children with a risk for serious systemic allergic reactions/anaphylaxis were to inject themselves with an active EAI (short needle and low dose), the proper use of EAIs may improve when true anaphylaxis occurs [37–39]. Another setting where patients can be trained or encouraged to use a live EAI is by having patients use live EAIs for epinephrine injection during reactions to oral food challenges.

## Intramuscular or subcutaneous injection?

Twenty years ago, Simons et al. concluded that intramuscular injection of epinephrine was superior to subcutaneous injection, due a significantly higher C_max_1 with IM injection. But as noted above, the true location of the injections in those studies were not confirmed. Recently, Duvauchelle et al. [11] showed that non-intended subcutaneous injection in women caused a non-significantly slower distribution than did intramuscular injection in men. In another recent study, Worm et al. [12] found paradoxically that injection, probably subcutaneous, led to a more rapid plasma concentration increase than intramuscular injection. The Turner et al. [13] study did not compare subcutaneous and intramuscular injection. Also, in migraine therapy, investigators actually prefer subcutaneous injection, using autoinjectors with short needles that require low pressure for spring release [40, 41]. The superiority of intramuscular over subcutaneous injection of EAIs has not been absolutely confirmed with epinephrine injections.

Therefore, it is essential to investigate the pharmacokinetics of epinephrine delivered in the muscle and the subcutaneous tissue using syringe and needle to confirm if intramuscular or subcutaneous injection is preferred.

## EAI studies during anaphylaxis

There are no randomized studies assessing epinephrine response during anaphylaxis episodes. Very little is known of the pharmacokinetics of epinephrine therapy during anaphylaxis. For ethical reasons, it is not possible to cause anaphylaxis in volunteers to study epinephrine responses. Recently, Moss et al. [42] proposed to study the impact of epinephrine on anaphylaxis in patients voluntarily participating in double blind, placebo controlled food challenges who have reactions. We suggest that patients participating in subcutaneous immunotherapy studies, receiving subcutaneous immunotherapy clinically, and patients receiving allergen provocation tests could be included in epinephrine studies as well.

## Conclusions

In conclusion, on reviewing the published data, intramuscular injection of epinephrine causes a C_max_1 with T_max_1 at approximately 5 min and a C_max_2 with T_max_2 after 30–50 min. Subcutaneous injection of epinephrine leads to a C_max_1 with T_max_1 about 15 min post injection as well as a C_max_2. This data supports that intramuscular delivery of epinephrine may be more effective than subcutaneous injections. But we believe that confirmatory pharmacokinetic studies should be performed with syringe and needle assuring injections of epinephrine into the intramuscular vs. subcutaneous tissues is necessary. The ultrasound localization of the bolus is essential to confirm proper and accurate delivery of the drug. Optimally, these studies should be completed while subjects are having allergic reactions. EAIs should be studied in a similar manner.

Finally, before new EAIs are developed, the validity of data documenting the need for intramuscular versus subcutaneous injection comparing different doses of epinephrine should be studied by independent researchers.

## Data Availability

Not applicable.
